# Clinical Application of Serum microRNAs in Atherosclerotic Coronary Artery Disease

**DOI:** 10.3390/jcm11226849

**Published:** 2022-11-20

**Authors:** Anna Kabłak-Ziembicka, Rafał Badacz, Tadeusz Przewłocki

**Affiliations:** 1 Department of Interventional Cardiology, Institute of Cardiology, Jagiellonian University Medical College, św. Anny 12, 31-007 Kraków, Poland; 2 Noninvasive Cardiovascular Laboratory, The John Paul II Hospital, Prądnicka 80, 31-202 Kraków, Poland; 3 Department of Interventional Cardiology, The John Paul II Hospital, Prądnicka 80, 31-202 Kraków, Poland; 4 Department of Cardiac and Vascular Diseases, Institute of Cardiology, Jagiellonian University Medical College, św. Anny 12, 31-007 Kraków, Poland

**Keywords:** acute coronary syndromes, atherosclerosis, cardiomyocytes, cardiovascular outcomes, endothelial cells, microRNAs, non-ST-segment elevation myocardial infarction, platelets, reperfusion injury, stable coronary artery disease, ST-segment elevation myocardial infarction, vascular smooth muscle cells

## Abstract

MicroRNAs (miRs) are promising diagnostic, prognostic and therapeutic biomolecules for atherosclerotic cardiovascular disease. Atherosclerotic occlusive disease concerns a large population of patients, carrying the highest incidence of fatal and non-fatal adverse events, such as myocardial infarction, ischemic stroke, and limb ischemia, worldwide. Consistently, miRs are involved in regulation and pathogenesis of atherosclerotic coronary artery disease (CAD), acute coronary syndromes (ACS), both with ST-segment (STEMI) and non-ST segment elevation myocardial infarctions (NSTEMI), as well as cardiac remodeling and fibrosis following ACS. However, the genetic and molecular mechanisms underlying adverse outcomes in CAD are multifactorial, and sometimes difficult to interpret for clinicians. Therefore, in the present review paper we have focused on the clinical meaning and the interpretation of various miRs findings, and their potential application in routine clinical practice.

## 1. Introduction

microRNAs (miRs) are endogenous non-coding single-stranded RNAs of approximately 20 nucleotides in length that negatively regulate post-transcriptional gene functions [1]. Since their discovery in the later years of the 20th century, miRs have become potential genetic biomarkers, among many other markers, for atherosclerotic cardiovascular disease [2,3,4]. Atherosclerotic occlusive disease concerns a large population, carrying the highest incidence of fatal and non-fatal adverse events worldwide, including myocardial infarction (MI), ischemic stroke, renal or limb ischemia [5,6,7]. Consistently, miRs has been shown to be involved in the regulation and pathogenesis of atherosclerotic stable coronary artery disease (CAD), acute coronary syndromes (ACS), both with ST-segment (STEMI) and non-ST segment elevation myocardial infarction (NSTEMI), as well as ischemia/reperfusion (I/R) injury, left ventricular remodeling (LVR) and fibrosis following ACS.

**Figure 1:**
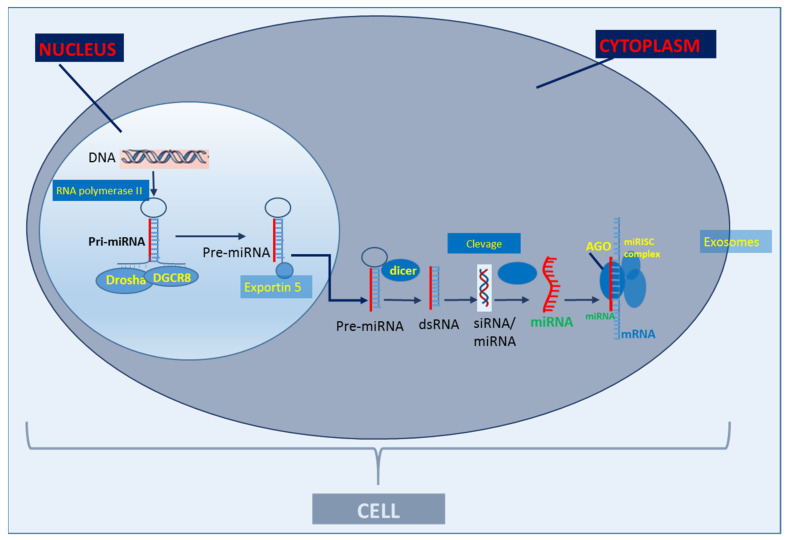
Basic scheme of classic miR biogenesis. The primary miRNA (Pri-miRNA) is produced in the cell nucleus through the transcription of a DNA strand mediated by RNA polymerase II. After transcription, Pri-miRNA is cleaved by the enzymatic complex DROSHA in a micro-RNA precursor (pre-miRNA). Pre-miRNA is exported to the cytoplasm by exportin-5 and cleaved by dicer (RNA degrading enzyme) and produces approximately 22 nucleotide RNA duplexes. A miRNA strand is transferred to the Argonaute complex (AGO), forming an RNA-induced silencing complex (RISC) and guides it to pair with the target messenger RNA (mRNA) through binding the miRNA seed sequence with the miRNA recognition site in the mRNA. miRs are secreted out of cells via exosomes (adapted from Creemers et al., 2012 [9]).

The genetic and molecular mechanisms underlying adverse outcomes in CAD are multifactorial, and sometimes difficult to interpret for clinicians. miRs have features that make them a potential diagnostic, prognostic and therapeutic target. As they regulate gene expression at the post-transcriptional level, usually by binding to the 3′-untranslated regions of target mRNAs, leading to the inhibited translation, and/or inducing degradation of the target mRNA (Figure 1) [8]. In this mechanism, a single miR can act on several or even hundreds of mRNAs. Furthermore, circulating miRs in serum are resistant to lysis. They show huge stability properties against RNase, e.g., by hiding themselves in various microvesicles (microparticles, apoptotic bodies, etc.). Thus, miRs keep the reins on all the major physiologic and pathophysiologic processes.

In a cardiovascular setting, the deprivation of vessel physiological processes, as a consequence of the degradation of responsible mRNAs, leads to the initiation of atherosclerosis [1,2,9,10,11,12]. The loss of vital mRNAs protecting against endothelial dysfunction, oxidative stress, low-grade inflammation, and many others, results in the promotion of atherosclerosis. The latter eventually leads to adverse atherosclerosis-related cardiovascular events [13,14]. Additionally, miRs play a fundamental role in either plaque destabilization or rupture, eventually triggering acute atherosclerotic ischemic events [1,2,10,11]. Eventually, they are important regulators of LVR, fibrosis, and I/R injury [10,11].

However, as the total number of all human miRs identified so far is over 2000 (www.mirbase.org, accessed on 22 August 2022), there is huge pressure to identify miRs that are crucial for adverse events incidence, which may result in production of antidotes against ‘bad’ miRs, or agonists to increase the expression of ‘good’ miRs [12].

Therefore, in the present review paper we have focused on the clinical meaning and the interpretation of various miRs findings, and their potential application in a routine clinical practice. We addressed the expression levels of circulating miRs in patients with stable CAD, ACS (STEMI/NSTEMI), I/R injury, and LVR following cardiac ischemia.

## 2. Atherosclerotic Stable CAD

Atherosclerotic CAD is estimated to cause death in 7–7.5 million people annually [6,15]. However, ACS and sudden death are responsible for 1.8 million deaths annually [16].

**Figure 2:**
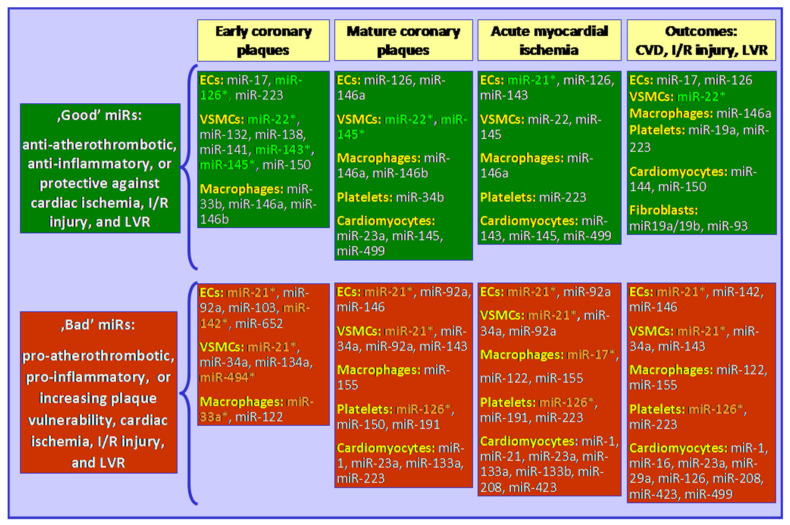
Selected miRs that have anti- or pro-atherothrombotic impacts on the development of atherosclerotic plaques, acute coronary syndrome, and/or post-myocardial ischemic outcomes. Please note that specific miRs are important at different stages of atherosclerosis. (1) At early stages of atherosclerosis (from fatty streaks undetectable with standard imaging tools up to intima-media complex thickening), miRs enriched in ECs, VSMCs and macrophages play a key role. Anti-atherothrombotic and anti-inflammatory effects are achieved through many signaling pathways, with a major regulatory role in lipids metabolism and inflammation. (2) At advanced stages of atherosclerosis, with development of mature plaques (easily detectable with various imaging tools), there is a struggle between miRs stabilizing plaque (e.g., increasing fibrous cap thickness, lowering inflammation, or lipids uptake), and miRs destabilizing plaque (e.g., thinning fibrous cap, enlarging lipid and necrotic core, or developing angiogenesis within the plaque). (3) At the stage of plaque rupture and thrombosis, there is a huge role for ECs-, platelets- and VSMCs/cardiomyocytes-derived miRs (such as miR-223 promoting ECs apoptosis, or miR-126 promoting angiogenesis). (4) After ACS, cardiovascular outcomes are associated with myocardial injury and cardiac fibrosis, in which cardiomyocytes-, VSMCs-, ECs-, and fibroblasts-derived miRs play a crucial role. Please note that some miRs can have dual roles, or opposing (conflicting) roles, in different cells (such as miR-223 which aggravates myocardial fibrosis but protects against hypoxia-induced apoptosis and oxidative stress). * miRs that may be used as therapeutic agents are noted in bold in clear green, while miRs that possess antagonists to neutralize their negative effects are in clear orange. CVD: cardiovascular death; ECs: endothelial cells; I/R: ischemia/reperfusion; LVR: left ventricular remodeling; VSMC: vascular smooth muscle cells.

A variety of miRs regulate multiple metabolic pathways responsible for atherosclerosis initiation, plaque formation, and finally plaque rupture [1,8,9,17]. As circulating miRs are origin-specific, they may be released from arterial endothelial cells (ECs: miR-17, 92a; -103; -126; -142; -652; etc.), cardiomyocytes (miR-1; -16; -23a; -133a/b; -208b; -423; -499, etc.), vascular smooth muscle cells (VSMCs: miR-22; -34a; -141; -143; -145; -132; -138; -150; -494; etc.), platelets (miR-34b; -34b; -126; -150; -191; -223, etc.), or macrophages (miR-33a/b; -146a/b; -155; -103; etc.) into a blood stream [1,17]. Some miRs are abundant in many cells, induced by shear stress miR-21 that is highly expressed in VSMCs, ECs, cardiac fibroblasts, cardiomyocytes, and platelets, and responsible for apoptosis and eNOS activity [18]. Among many candidate miRs, some are of special interest as potential diagnostic markers for significant CAD, ACS, and LVR, prognostic markers for cardiovascular events or even a promising therapeutic target in atherosclerosis [1,17,19]. Specific miRs addressed in this review, with the inclusion of their anti-, and/or pro-atherothrombotic regulatory functions, are presented in Figure 2.

### 2.1. miRs Diagnostic for CAD

In patients suspected of chronic coronary insufficiency, some of miRs may help to discriminate patients with or without significant CAD (Table 1). For example, the discriminating power of circulating miR-145, miR-182, miR-205, and miR-133a was found to be highly predictive of stable CAD with receiver operating characteristic curves (ROC) and area under the curve (AUC) values above 0.80 [20]. Cardiac muscle–enriched miRs, such as miR-133a, miR-134, miR-145, miR-122, and miR-370 are associated with the presence of CAD [19,21]. EC-enriched circulating levels of miR-126, miR-17, miR-92a and macrophage inflammation-associated miR-155 were significantly reduced in patients with CAD compared with healthy controls [21]. Similarly, the smooth muscle–enriched miR-145 was significantly reduced in CAD [22]. However, when patients presented with unstable angina and chest pain corresponding to vulnerable coronary plaques on a coronary angiography, several vascular and inflammation-associated miRs, previously reported as down-regulated in stable CAD, such as miR-21, miR-17, miR-20a, and miR-92a, were found to be up-regulated in unstable angina [21]. Thus, the role and clinical significance of individual miRs can be subject to variability depending on the stage of atherosclerotic disease, and its stable or unstable presentation.

#### 2.1.1. Platelets Activity

Although platelets do not contain a nucleus and genomic DNA, they have been shown to contain subcellular organelles, as well as a small amount of poly(A) + RNA from their megakaryocyte progenitor cells [23,24]. It is estimated that between 15% and 32% of protein-coding genes are represented in the form of mRNAs in platelets [23].

There are some platelets-derived miRs that play a key role in thrombosis and hemostasis [25,26,27]. Some of them are responsible for platelet activation and are influenced by antiplatelet therapy [25,26,27,28,29,30,31]. Platelet activity, responsible for blood clotting, might be modified by miR-34b, miR-126-5p, miR-150, miR-223-3p, and miR-191 (Table 1) [25,26,27,28,29,30,31].

miR-34b-3p has be associated with thromboxane-mediated platelet aggregation through the cyclooxygenase 1 (COX-1) receptor. COX-1 is the therapeutic target of aspirin, which irreversibly binds and inactivates the enzyme [28]. Inhibition of miR-34b-3p in megakaryocytes increased their viability and decreased the expression of thromboxane synthase and thromboxane B2, a stable metabolite of TxA2 [25]. Thus, miR-34b-3p may facilitate the antiplatelet efficiency of aspirin through inhibiting thromboxane synthase [28].

miR-223, one of the most abundant miRNAs in megakaryocytes and platelets, targets the P2Y12 receptor [26,29]. miR-223-deficient mice form larger thrombi and have a delayed clot retraction compared to wild type mice [27]. Platelets of miR-223-deficient mice display increased aggregation in response to thrombin and collagen [27]. Some miRs (miR-126-3p and miR-223-3p) play a role in platelets activation in ACS, and their expression levels are modified by antiplatelets therapy with aspirin and P2Y12 receptor inhibitors [24,30]. Antiplatelet therapy significantly reduces levels of miR-223, miR-191, miR-126 and miR-150, that decreases platelet inhibition [28]. In line with this, an up-regulation of miR-126 is associated with a higher platelet susceptibility to aggregate, while administration of the antagomir against miR-126-3p reduces platelets aggregation (Table 1) [29].

Due to numerous limitations of the currently used platelet function test, studies position miRs as promising candidates to assess the platelet response to COX-1 and P2Y12 inhibitors, as they are stable in biological samples. However, their low levels may impose difficulties in their quantification [30,31].

#### 2.1.2. Vascular Smooth Muscle Cells

VSMC proliferation plays a critical role in atherosclerosis [32]. At the beginning of atherothrombotic process, irregular VSMC proliferation promotes plaque formation, but in advanced plaques VSMCs are beneficial, promoting stability and preventing rupture of the fibrous cap [32,33].

VSMCs have two states: contractile or synthetic/proliferative [34,35,36]. The first is associated with maintaining vessels tone and elasticity, while the second characterizes arterial stiffening and promotes atherosclerotic growth [34,35]. Increasing arterial stiffness is a well-known risk factor for adverse cardiovascular events, including cardiovascular death (CVD) [37,38]. High expression of miR-22, miR-124, miR-132-3p, miR-138-5p, miR-141-3p, miR-145, and miR-150-5p is needed for the contractile phenotype of VSMCs [39]. Of those, an increased expression of miR-22, miR-124 and miR-145 can reverse VSMC phenotypic switching, favoring the contractile phenotype (Table 1) [40,41]. In models adding miR-145, or miR-22, the contractile function of VSMCs can be restored [40,41]. Importantly, miR-145 is responsible for the increase in plaque collagen content and enlarging the fibrous cap area, in line with reducing the necrotic core area [42,43]. In the study by Gao et al., reduced plasma miRNA-145 levels correlated with an increase in CAD severity (a SYNTAX score) [44].

Wang et al. demonstrated that the sustained release of miR-22 enhanced the contractile phenotype of VSMCs without interfering with the proliferation of ECs [45]. Moreover, miR-22 showed therapeutic potential against restenosis, as the miR-22-coated stents showed a significant capability to inhibit in-stent restenosis in minipigs (an animal study) (Table 1) [45]. Although more controversial, a similar role was proposed for anti-miR-21-eluting stents in rats, as miR-21 is abnormally expressed in patients with coronary restenosis [46,47]. These findings are very relevant in everyday clinical practice, as restenosis following endovascular interventions with stent implantation is a serious drawback [48]. It seems that miRs could help to overcome this clinical problem.

On the contrary, an increased expression of miR-34a, while a decreased expression of miR-143 promotes vascular senescence of VSMCs [34,49]. In a study enrolling 203 patients with stable CAD and 100 controls, miR-34a showed good diagnostic value for CAD presence with an AUC of 0.899 (*p* < 0.001), and was associated with a Gensini score in CAD patients (*p* < 0.001) [50].

#### 2.1.3. Arterial Endothelial Cells

Vascular endothelium injury and dysfunction are pivotal in the atherosclerotic process and comprise components such as: inflammation, cells infiltration, EC apoptosis and neointimal formation. EC miRs are regulated upon shear stress and disturb laminar flow in the arteries [51]. ECs harbor large amounts of regulatory miRs, with the highest expression for miR-21-5p, miR-126-3p, and the family and clusters of let-7 miRNA, miR-17-92, and miR-221/222 [51,52,53].

Among them, miR-126 is a crucial regulator of atherosclerosis [53,54,55,56,57,58]. miR-126-5p promotes regenerative proliferation of ECs, and limits atherosclerosis [54,55,56]. Up-regulation of miR-126 is also required for angiogenesis during organismal development or the repair of injured arterial vasculature [53]. In patients with a down-regulated expression of miR-126, more severe and complex CAD has been observed [57,58]. In line with this, Wang et al. observed that the miR-126 expression was significantly down-regulated in CAD patients compared to control subjects, but up-regulated in patients presenting with ACS compared to the controls [58].

In contrast, increased miR-652-3p expression levels promoted atherosclerosis through the inhibition of the EC regeneration and repair following mechanical injury [59]. The same was observed for miR-142-3p in which high expression levels increased EC apoptosis and atherosclerotic development by up-regulating the expression of Rictor and activating the Akt/eNOS signaling pathway [60]. Treatment with the miR-142-3p antagomir attenuated endothelial apoptosis and retarded the progression of atherosclerosis in the aorta of ApoE-/- mice [60].

In addition, miR-103-3p stimulated inflammatory activation, and uptake of oxidized LDL-cholesterol promoting atherosclerotic growth [61,62]. Thus, a reduction in miR-103 levels, also results in the reduction in atherosclerosis and endothelial inflammation [61]. Similarly, miR-92a-3p is up-regulated in ECs in response to shear and oxidative stress, and oxidized LDL [63]. Overexpression of miR-92a promotes ox-LDL (including malondialdehyde-LDL)-induced apoptosis [64]. The latter was associated with high-risk plaques in the coronary arteries despite statin treatment [65]. Up-regulation of miR-92a and miR-486 could discriminate between patients with stable and vulnerable CAD [66]. Another, miR involved in lipid metabolism is miR-122 that is increased in the plasma of patients with significant CAD [67]. In 255 hyperlipidemia patients with or without CAD and 100 control patients with normal blood lipid levels, miR-122 and miR-370 levels were positively correlated with the severity of CAD quantified by the Gensini score [67]. Finally, miR-17-5p was highly expressed in ECs, but low in VSMCs. There was a negative correlation between miR-17 expression levels and CAD severity [68]. However, high miR-17 expression led to the inhibition of angiogenesis, which is desirable after ACS [69].

#### 2.1.4. Macrophages

Macrophages contain several miRs, including, most prevalently, miR-146a-5p, miR-10a-5p, let-7 family members, miR-21a-5p, and miR-155-5p [52,70,71]. They are responsible for atherosclerotic progression, the uptake of lipoproteins, self-transformation to foam cells and their subsequent apoptosis, cholesterol crystal deposition and necrotic core formation, and mature lesions with a thrombogenic core [52]. Macrophages are involved in cholesterol homeostasis and are regulated by several miRs involved in lipid metabolism. In cholesterol homeostasis, inhibition by miR-33a lowers plasma cholesterol and reduces plaque burden, while miR-33b the increases level of plasma HDL-cholesterol [72].

Some miRs that take part in atherosclerotic lesion progression are activated by lipopolysaccharides [52]. One of them, miR-155-5p has an ambiguous role in atherosclerosis [71,73,74,75,76]. At the early stages of atherosclerosis, miR-155 reduce macrophage proliferation [73], while in advanced atherosclerotic lesions it promotes further formation of atherosclerotic lesions through mediation of pro-inflammatory processes [74]. In addition, miR-155 increases glucagon-like peptide 1 (GLP-1), which inhibits glucagon production, reduces adipose tissue, and improves glucose metabolism, but at the same time miR-155 is associated with increased lipid levels [75]. Dysregulated miR-155 levels play a crucial role in the pathogenesis of diabetes mellitus [76]. This ambiguous action of miR-155 limits its clinical application. Research studies using intravascular ultrasonography (IVUS) and optimal coherence therapy (OCT) have demonstrated that coronary lesions can be found at various growing stages at the same time [77]. In an individual patient, some atherosclerotic lesions can be limited to fatty streaks, while others can have features of a necrotic core, or even rupture, and they all are regulated by miRs [78].

Lipopolysaccharide-induced macrophage activation through the expression of miR-146b is another mechanism of atherosclerotic evolution [79,80,81,82,83]. miR-146a antagonizes the pro-inflammatory effects of miR-155 [79,80]. miR-146a has a critical role as an anti-inflammatory and athero-protective agent, constituting a ‘brake’ for inflammation through the inhibition of IL-6, IL-1β, IL-8 and TNF-α [81]. It inhibits oxidized LDL-induced lipid accumulation and inflammatory response via targeting of Toll-like receptor 4 [80]. miR-146a plays a major role in thrombo-inflammation, i.e., thrombosis associated with an inflammatory process [81]. Its rise after an MI is cardioprotective as it suppresses apoptosis, the inflammatory response, and fibrosis [82]. In contrast, low expression levels of miR-146a facilitate an increase in infarct size, apoptosis and I/R injury following ACS [83].

#### 2.1.5. Cardiomyocytes

miRs contained within cardiomyocytes are released into blood stream as a result of cardiac cell ischemia, that leads to cell damage [84,85]. Among cardiomyocyte-enriched miRs, some deserve special attention, such as miR-1, miR-133a, miR-133b, miR-145, miR-208b, miR-223, and miR-499, and they all (if detected in serum) are considered markers of myocardial damage [1,12,19,84,85,86,87,88,89,90,91,92,93,94].

Their clinical significance results from the diagnostic properties to distinguish healthy individuals from those with significant CAD, unstable angina, or ACS (STEMI and NSTEMI) (Table 1) [1,12,19,19,84,85]. In a study by Guo et al., researchers analyzed a group of 300 patients with stable CAD compared to 100 healthy control individuals, the level of circulating miR-223 was highly predictive of the severity of CAD with an AUC of 0.933 [86]. In a study by Zhu et al., among the five examined miRs (miR-92a, miR-133a, miR-133b, miR-125b, and miR-21), only miR-133a was significantly increased in CAD patients, compared to the healthy control patients; however, at a low AUC value (0.597) [87].

In a study by Abdallah et al., researchers enrolled 73 patients with stable CAD and compared them to 73 control patients. miR-133a, miR-155 and miR-208a were down-regulated, while miR-182, miR-145, miR-21, miR-126, miR-200b, miR-146a, miR-205, miR-135b, miR-196b, and miR-223 were significantly up-regulated [88]. In this study, miR-133a, miR-182, miR-145, and miR-205 showed satisfactory diagnostic levels with AUC values above 0.8 [88].

Navickas et al. reviewed 19 studies to identify miRs that could be used as biomarkers in plasma/serum to diagnose patients with atherosclerosis, significant CAD and/or ACS [19]. Out of analyzed miRs, miR-1, miR-208, and miR-133a were observed as significant serum biomarkers of acute chest pain associated with cardiac ischemia [19]. While de Rosa et al. observed that the muscle-enriched miR-499 (20-fold), miR-133a (11-fold), and miR-208a (5-fold) were significantly elevated in the aorta of troponin-positive ACS patients, compared to patients with stable CAD [89].

**Table 1:** Summary of the most clinically utile miRs in atherosclerotic coronary artery disease, with the inclusion of the miRs role, place of origin, expression levels, and targets.

Postulated Role	microRNA	Down- vs. Up- Regulated	Diagnostic/Therapeutic	Reference
	Expressed in many cells			
Highly expressed in VSMCs, ECs, cardiac fibroblasts, cardiomyocytes, and platelet apoptosis and eNOS activity	miR-21-5p	Up	D, up-regulated in CAD patients compared to controls (AUC: 0.767, *p* < 0.001)	Abdallah H.Y., 2022 [88]
	Platelets			
Humans: collagen-induced platelet aggregation Mice: expression of the P2Y12 receptor	miR-126-3p	Up	D, monitors P2Y12 inhibition	Kaudewitz D., 2016 [26]
Responsive to antiplatelet therapy	miR-126-3p	Up	T, an antagomir against miR-126-3p reduces platelets aggregation	Kaudewitz D., 2016 [26]
Marker of platelet activation, that targets the COX-1 receptor through the regulation of TXS	miR-34b-3p	Up	D, miR-34b-3p may facilitate the antiplatelet efficiency of aspirin through inhibiting TXS	Liu W.W., 2009 [28]
Marker of response to clopidogrel, that targets the P2Y12 receptor	miR-223-3p	Down	D, high on-clopidogrel platelet reactivity	Shi R., 2016 [29]
miRs released by platelets, that are responsive to antiplatelet therapy	miR-126 miR-150 miR-191 miR-223	Up Up Up Up	D, antiplatelet therapy significantly reduces their levels	Czajka P., 2021 [31]
	VSMCs			
High expression is needed to maintain a contractile phenotype of VSMCs	miR-22	Down	T, a stent with an miR-22 coating showed significant capability to inhibit in-stent restenosis (an animal study)	Yang F., 2018 [40]
Mitigates atherosclerosis, VSMCs contractility, increases fibrous cap area, and reduces the necrotic core area	miR-145	Down Down	T, delivery of miR-145 may limit atherosclerotic plaque growth, and restore contractile levels in VSMCs	Patel N., 2022 [42]
Down-regulation of miR-145 plays a critical role in the pathogenesis of atherosclerotic plaques, and neointimal hyperplasia	miR-145	Down	D, reduced plasma miR-145 levels correlate with an increase in CAD severity (SYNTAX score)	Gao H., 2015 [44]
Induces VSMC senescence, promotes the expression of age-associated pro-inflammatory secretory factors, and increases the binding capacity of ox-LDL to macrophages	miR-34a	Up	D, increased expression in CAD, compared to healthy controls (AUC: 0.899, *p* < 0.001), associated with Gensini score (*p* < 0.001)	Li H., 2022 [50]
	Arterial endothelial cells			
Plays a crucial anti-atherogenic role by regulating the function of ECs and enhancing endothelial repair	miR-126-3p	Down	D, reduced expression is associated with more severe and complex CAD	Li H., 2016 [57]
Decreases size of atherosclerotic lesions, alleviates ox-LDL-induced EC injury	miR-126-3p	Down Up	D, decreased expression in CAD patients, compared to healthy controls, but up-regulated in ACS	Wang X., 2017, [58]
Induces EC apoptosis, development of atherosclerosis	miR-142-3p	Up	T, down-regulation of miR-142-3p suppresses ECs apoptosis	Qin, B., 2018 [60]
Induces apoptosis and oxidative stress, and is pro-atherosclerotic	miR-92a-3p miR-486	Up Up	D, discriminate between stable and vulnerable CAD	Niculescu L.S., 2015 [66]
Lipid metabolism	miR-122	Up	D, increased in CAD patients, and with CAD severity (Gensini score)	Gao W., 2012 [67]
Recovery of ischemic tissue	miR-17	Down	D, reduced expression is associated with more severe and complex CAD	Chen J., 2015 [68]
Rate of apoptosis in ECs	miR-17-5p	Up	T, inhibition of miR-17 suppresses apoptosis, hence, decreases infarct size area, and improves microcirculation of heart tissue, decreasing heart failure symptoms	Yang S., 2018 [69]
	Macrophages			
Regulator of cholesterol and fatty acid homeostasis, reverse cholesterol transport, increases HDL-cholesterol level	miR-33a miR-33b	Up Up	T, inhibition of miR-33a facilitates atherosclerotic regression	Price N.L., 2017 [72]
Inhibits oxidized LDL-induced lipid accumulation and inflammatory response	miR-146a	Up	D, patients with stable CAD had 3.62-fold higher expression level, compared to controls (AUC:0.767)	Abdallah H.Y., 2022 [88]
Reduced in diabetics, has a role in lipid metabolism	miR-155	Down	D, patients with stable CAD had 1.89-fold lower expression level, compared to controls (AUC:0.767)	Abdallah H.Y., 2022 [88]
	Cardiomyocytes			
Indicates myocardial damage	miR-223-5p	Up	D, increased expression, compared to healthy control group, with a AUC of 0.933 for predicting CAD severity	Guo J.F., 2018 [86]
Suppresses EC proliferation rate, viability, and migration activity involved in heart development, and indicates myocardial damage	miR-133a	Up	D, increased expression in CAD, compared to healthy controls, but with a low AUC of 0.597 correlates with Gensini score of CAD severity (r = 0.303, *p* = 0.007)	Zhu L., 2017 [87]
Indicates myocardial damage Indicates myocardial damage Fibrous cap increase Indicates myocardial damage Fibrous cap thinning Plaque neovascularization Indicates myocardial damage	miR-133a miR-182 miR-145 miR-205 miR-208a miR-21 miR-126 miR-223	Down Up Up Up Down Up Up Up	CAD vs. controls: AUC: 0.863, *p* < 0.001 AUC: 0.959, *p* < 0.001 AUC: 0.836, *p* < 0.001 AUC: 0.959, *p* < 0.001 AUC: 0.616, *p* = 0.015 AUC: 0.767, *p* < 0.001 AUC: 0.767, *p* < 0.001 AUC: 0.616, *p* = 0.015	Abdallah H.Y., 2022 [88]
Induces angiogenesis and myocardial damage Indicate myocardial damage	miR-1 miR-133a	Up Up	D, increased expression, compared to a healthy control group	Kuwabara Y., 2011 [90]
Expressed in myocardial cells	miR-23a	Up	D, up-regulated, positive correlation with CAD severity	Lu H.Q., 2013 [91]
Cardiac myofibroblast differentiation, smooth muscle cell modulator, increases fibrous cap area, reduces necrotic core	miR-145	Down	D, reduced in patients with cardiac ischemia	Zhang M., 2017 [92]
Indicates myocardial damage, cardiac hypertrophy Protects against H_2_O_2_-induced apoptosis	miR-208b miR-499	Up Up	D, independent predictors of a high SYNTAX score miR-208b: AUC: 0.775, *p* < 0.001 miR-499: AUC: 0.713, *p* < 0.001	Wang W., 2019 [93]
Increases foam cell formation	miR-23a	Up	D, correlates with CAD severity (Gensini score)	Wang S., 2016 [94]

AUC: area under the curve; D: diagnostic; miR: microRNA, CAD: coronary artery disease; ECs: endothelial cells; T: therapeutic approach; TXS: thromboxane synthase; VSMC: vascular smooth muscle cells.

Of note, decreased miR-27b and miR-146 expression levels are postulated to be associated with a higher severity of coronary, lower extremity, and carotid atherosclerosis [95]. These miRs presented an AUC of ≥0.75 for predicting polyvascular atherosclerosis involving the three territories [95]. Polyvascular atherosclerosis is a major clinical issue, both due to its relatively high prevalence as well as increased risk of major adverse coronary and carotid events [96,97,98]. As miRs are abundantly present in a remarkably stable form and can be detected in peripheral circulation, they are natural biomarkers of atherosclerosis at any stage of its evolution, as well as the indicators of plaque vulnerability and acute ischemia [99,100].

## 3. Acute Coronary Syndrome

### 3.1. miRs Diagnostic in ACS

ACS is a result of the interplay between coronary artery in situ thrombus formation, vulnerable plaque features such as a lipid or a necrotic core, myocardial necrosis followed by fibrosis. As previously evidenced, several miRs follow the same kinetics as highly sensitive cardiac troponins (cTn), since they derive from myocardial necrosis [1,19].

For miRs to be considered as diagnostic markers for ACS, they must be quickly released, optimally preceding typical cardiac markers of myocardial necrosis, such as cTn, creatine kinase MB. Then, the potential miR must be characterized by high sensitivity and specificity for ACS, preferably with a power assessed with AUCs above 0.9. They should well-differentiate patients with ACS from those with stable CAD and healthy individuals (Table 2).

There is growing evidence that these criteria are fulfilled for miR-1, miR-133a and miR-133b that may have an advantage over other miRs, as their peak concentration has been documented to anticipate the peak cTn concentration, even at 2.5 h after the onset of chest pain [101]. Plasma miR-1 levels were shown to be significantly up-regulated in 93 ACS patients on admission compared to 66 healthy controls, and this decreased to similar level observed in healthy volunteers on discharge [102]. In a study by Long et al., circulating miR-1 and miR-126 in ACS patients significantly differed compared to healthy adults, with a peak change at 4 and 8 h since symptom onset, then the fold change gradually decreased over time [103]. Both miR-1 and miR-126 showed high sensitivity and specificity for ACS (Table 2). In line with this, Kazimierczyk et al. demonstrated that the concentration levels of serum miR-1 and miR-126 were higher in ACS patients on admission, compared to the controls [104]. Moreover, miR-1 correlated positively with the maximal cTn concentration (r = 0.59, *p* = 0.02), and negatively with the left ventricular ejection fraction (LVEF) (r = −0.76, *p* = 0.0004) [104]. Of note, in the work by Wang et al., and Widera et al., higher expression levels of miR-1, miR-133a, and miR-208a were found in patients with cardiac ischemia compared to healthy subjects [105,106]. In addition, Wang et al. and Zhang et al. found good accuracy for miR-499 for the early diagnosis of ACS [105,107]. Conversely, in a study by He et al., the AUC value of miR-126-3p performed better (AUC: 0.992, *p* < 0.001), compared to cTn (AUC 0.787, *p* < 0.001), and creatine kinase MB (AUC 0.863, *p* < 0.001) [108]. Additionally, Gidlöf et al. observed in 25 patients with STEMI an abrupt increase in miR-1, miR-133a, miR-208b and miR-499-5p with a peak within 12 h from the onset of chest pain. Moreover, expression levels of miR-208b correlated with peak cTn and the left ventricular ejection fraction [109]. In line with this, Su et al. identified miR-1 as an early marker of ACS with a similar diagnostic accuracy to cTn [110].

Xue et al. proposed a different set of miRs in the diagnosis of ACS [111]. In this study, the expression levels of plasma miR-17-5p, miR-126-5p, and miR-145-3p showed considerable diagnostic efficiency for ACS, as individual measurement, and in the combination [111]. Horvath et al. observed up-regulated levels of miR-24, miR-146a, miR-145, miR-151-3p, miR-323p, and miR-331 in STEMI compared to patients with stable CAD and healthy individuals [112]. Similarly, miR-223 and miR-191, markers of platelet activation, showed higher expression levels in patients with STEMI compared to healthy controls and stable CAD patients, indicating the presence of intracoronary thrombus as the trigger for ACS [112]. The ROC analysis confirmed the suitability of miR-331 and miR-151-3p as early biomarkers of STEMI (in a median of 2.25 hours since the onset of chest pain), while the markers of myocardial necrosis were still negative at the time of sampling [112].

Conflicting data presented by Meng et al. found decreased plasma levels of miR-143 and miR-145 in patients presenting with ACS compared to controls [113]. Both miRs were negatively correlated with Gensini score, and they showed good predictive value for the onset of ACS (miR-143: OR 0.087, 95% CI 0.026–0.384, *p* = 0.019, and miR-145: OR 0.179, 95% CI 0.08–0.399, *p* < 0.001) [113]. In line with this, data from the REGICOR registry comparing 500 samples from ACS patients matched with 500 samples from healthy controls showed that miR-143 was significantly associated with time-to-ACS (HR 0.56, 95% CI 0.38–0.82), *p* = 0.003) [114].

Less evidence exists for miR-23a-3p, although in a study by Bukauskas et al. miR-23a-3p showed relatively high predictive value for STEMI (AUC 0.806, 95% CI 0.694–0.917), compared to healthy individuals, and provided information on the 1-year mortality according to the GRACE and APACHE scales (*p* = 0.045, log-rank tests) [115]. Zhang et al. found that plasma levels of miR-21 were significantly higher in patients with AMI or angina compared to the controls. They also found a significant correlation between miR-21 and clinically established markers, including cTn and creatine kinase MB (*p* < 0.001) [116].

An interesting approach was presented by Kayvanpour et al. [117]. In their study, the authors developed a neural network model which incorporated 34 validated ACS miRs, showing excellent classification results with an accuracy of 0.96 (95% CI 0.96–0.97), sensitivity of 0.95, specificity of 0.96 and AUC of 0.99, compared to the one-point cTn value (accuracy of 0.89, sensitivity of 0.82, specificity of 0.96, and AUC of 0.96) [117].

#### Differences between STEMI and NSTEMI

Although, many studies have enrolled patient with ACS, including both STEMI and NSTEMI patients [1,19,102,103,105,106,107,108], some studies have addressed miR expression levels with respect to the type of ACS, Table 2 [100,101,102,103,104,105,106,107,108,109,110,111,112,113,114,115,118,119,120,121,122,123]. Most evidence has been reported on STEMI and miRs, but much less for NSTEMI.

In a study by Bukauskas et al., higher expression levels were found for miR-23a, miR-30d, miR-146 in STEMI patients compared to healthy participants [115]. In a study by Biener et al. enrolling 137 NSTEMI patients and 905 patients admitted with chest pain (after exclusion of STEMI), higher expression levels were found for five miRs (miR-29a, miR-92a, miR-126, miR-132, and miR-133) [119]. However, the AUCs were disappointingly low, ranging between 0.577 and 0.656 for individual miRs, and 0.662 for the panel of the most predictive miRs [110]. Furthermore, in this study cTn changes had a higher predictive value for NSTEMI than miRs [110].

In a study by Liu et al. including 145 NSTEMI patients and 30 control subjects, the expression levels of miR-1, miR-133, miR-208, and miR-499 were analyzed [120]. The authors found that three out of the four analyzed miRs (miR-133, miR-208 and miR-499) demonstrated superior diagnostic accuracy than cTn (AUC: 0.778), Table 2 [120]. In contrast, Zhelankin et al. found increased plasma levels of miR-146a-5p and miR-21-5p was a general ACS circulating biomarkers and lower levels of miR-17-5p was a general biomarker of CAD [121]. In a study by Gacoń et al., the increased expression level of miR-134 in STEMI compared to NSTEMI patients was observed [122]. Interestingly in that study, patients with occluded compared with patient infarct-related coronary artery thrombosis had higher levels of miR-133a (fold change: 7.00), miR-133b (4.57), miR-34a (5.50), and miR-124 (2.55), providing a significant signature for acute plaque rupture with subsequent coronary artery thrombosis, irrespective of ACS type [122]. Thus, miR expression might indicate subjects in which a coronary angiography should be performed without further delay due to artery occlusion, and the increased risk of myocardial injury.

In 62 patients with unstable angina, Zhang et al. found that a decrease in miR-223 levels was the only independent predictor for platelet reactivity index-determined lower responders [123].

**Table 2:** microRNAs that are potentially diagnostic in acute coronary syndromes.

Study Groups, N of Participants	microRNA	Down vs. Up-Regulated	Rationale for Use of Individual microRNA	AUC, or OR (95% CI), *p*-Value	Reference
STEMI/NSTEMI, 93 Healthy Controls, 66	miR-1	Up	D, early marker, up-regulated expression, compared to healthy control group	AUC: 0.774, *p* < 0.001	Ai J., 2010 [102]
STEMI/NSTEMI, 17 Healthy Controls, 25	miR-1 miR-126-3p	Up Down	D, early markers, changed expressions, compared to healthy control group	AUC: 0.92, *p* = 0.001 AUC: 0.860, *p* = 0.01	Long G., 2012 [103]
STEMI/NSTEMI, 33 Healthy Controls, 33	miR-1 miR-133a miR-208a miR-499	Up Up Up Up	D, early markers, increased expressions, compared to healthy control group	AUC: 0.850, *p* = 0.001 AUC: 0.870, *p* = 0.01 AUC: 0.970, *p* = 0.001 AUC: 0.820, *p* = 0.01	Wang G.K., 2010 [105]
STEMI/NSTEMI, 142 Non-ACS chest pain, 100 Healthy Controls, 85	miR-499	Up	D, early marker of ACS, 1 h after onset of chest pain, correlated with CK-MB level and cTn, but not superior to cTn (AUC: 0.90)	AUC: 0.860, *p* < 0.001	Zhang L., 2015 [107]
STEMI/NSTEMI, 27 Healthy Controls, 30	miR-126-3p	Down	D, early marker, diagnostic effect superior to cTn (AUC 0.787) and CK-MB (AUC 0.863)	AUC: 0.992, *p* < 0.001	He Y., 2017 [108]
STEMI, 25 Healthy Controls, 11	miR-1 miR-133a miR-208b miR-499-5p	Up Up Up Up	D, with a peak within 12 h from onset of chest pain, expression levels of miR-208b correlated with peak cTn and the LV ejection fraction	AUC: 0.980, *p* < 0.001 AUC: 0.859, *p* = 0.007 AUC: 1.000, *p* < 0.001 AUC: 0.989, *p* < 0.001	Gidlöf O.; 2011 [109]
STEMI, 106 NSTEMI, 68 Non-ACS chest pain, 163	miR-1	Up	D, early marker of ACS within 3 h since onset of chest pain, similar AUC to cTn (AUC: 0.862, *p* < 0.001)	AUC: 0.863, *p* < 0.001	Su T., 2020 [110]
STEMI, 15 NSTEMI, 14 Healthy Controls, 21	miR-17-5p miR-126-5p miR-145-3p	Up Up Up	D, within 4 h after the onset of chest pain	AUC: 0.857, *p* < 0.001 AUC: 0.802, *p* < 0.001 AUC: 0.720, *p* = 0.01	Xue S., 2019 [111]
STEMI, 20 CAD, 20 Healthy Controls, 20	miR-151-3p	Up	D, proceeded release of necrotic markers, increased expression, compared to healthy controls and stable CAD	STEMI vs. controls: AUC: 0.758, *p* = 0.005 STEMI vs. CAD AUC: 0.754, *p* = 0.006	Horvath M., 2020 [112]
STEMI, 20 CAD, 20 Healthy Controls, 20	miR-331	Up	D, proceeded release of necrotic markers, increased expression, compared to healthy and stable CAD	STEMI vs. controls: AUC: 0.790, *p* = 0.002 STEMI vs. CAD AUC: 0.773, *p* = 0.003	Horvath M., 2020 [112]
STEMI/NSTEMI, 78 Unstable angina, 201 Healthy Controls, 65	miR-143 miR-145	Down Down	D, down-regulated compared to controls, good predictive value for the onset of ACS	0.087 (0.026–0.384), *p* = 0.019 0.179 (0.08–0.399), *p* < 0.001	Meng L., 2022 [113]
ACS, 500 Healthy Controls, 500	miR-143	Down	D, down-regulated compared to controls, good predictive value for the onset of ACS	0.56 (0.38–0.82), *p* = 0.003	Dégano I.R., 2020 [114]
STEMI, 62 Healthy Controls, 26	miR-23a-3p miR-30d-5p miR-146a-5p	Down Down Down	D, for STEMI vs. healthy controls; *p*, correlated with GRACE and APACHE scores of in-hospital mortality, and 1-month survival D, for STEMI vs. healthy controls D, for STEMI vs. healthy controls	AUC: 0.806, *p* < 0.05 *p* = 0.045 (log-rank tests) AUC 0.745, *p* <0.05 AUC 0.800, *p* < 0.05	Bukauskas T., 2019 [115]
NSTEMI, 137 Chest pain *, 905	miR-126 miR-133 miR-134	Up Up Up	D, diagnostic for NSTEMI, but not superior to cTn (AUC: 0.937)	AUC: 0.578, *p* = 0.003 AUC: 0.656, *p* < 0.001 AUC: 0.506, *p* = 0.032	Biener M., 2021 [119]
NSTEMI, 145 Healthy Controls, 30	miR-1 miR-133 miR-208 miR-499	Up Up Up Up	D, for NSTEMI vs. healthy controls, miR-133, miR-208 and miR-499 superior to cTn (AUC: 0.778)	AUC: 0.772, *p* < 0.05 AUC: 0.928, *p* < 0.05 AUC: 0.994, *p* < 0.05 AUC: 0.994, *p* < 0.05	Liu G., 2018 [120]
STEMI, 16 NSTEMI, 27	miR-134 miR-134 miR-124 miR-133b	Up Up Up Up	D, for STEMI, but not superior to cTn D, for occluded IRA D, for occluded IRA D, for occluded IRA	AUC: 0.725, *p* = 0.002 AUC: 0.686, *p* = 0.016 AUC: 0.787, *p* < 0.001 AUC: 0.704, *p* = 0.006	Gacoń J., 2016 [122]
NSTEMI	miR-223-3p	Down	Marker of response to clopidogrel, targets P2Y12 receptor D, lower response to clopidogrel in NSTEMI	0.111, (0.018–0.692), *p* = 0.019	Zhang Y.Y., 2014 [123]

* excluded patients with STEMI; ACS: acute coronary syndrome; AUC: area under the curve; CAD: coronary artery disease; CI: confidence interval; CK-MB: creatine kinase MB; cTn: cardiac troponin, D: diagnostic; IRA: infarct related artery; LV: left ventricle; miR: microRNA, NSTEMI: non-ST elevation myocardial infarction, OR: odds ratio; STEMI: ST-elevation myocardial infarction.

In summary, miR-1, miR-133, and miR-499 have the greatest potential for diagnostic biomarkers of ACS, as they are detected in blood samples before cTns. A meta-analysis of twenty-six studies enrolling in total 1973 ACS patients and 1236 healthy controls, indicated miR-1, miR-133 and miR-499 to have the highest value as diagnostic biomarkers of ACS [115]. The pooled sensitivity for miR-1 in the diagnosis of ACS was 70% (95% CI: 0.66–0.74), specificity: 81% (95% CI: 0.78–0.85), and AUC of 84%. The values for miR-133 were 82% (95%CI: 0.77–0.86), 87% (95%CI: 0.82–0.90) and 92.9% respectively, while for miR-499 were 80% (0.77–0.83), 89% (0.86–0.92) and 89.8%, respectively [124].

However, as time since ACS diagnosis to coronary artery revascularization is critical in post-myocardial injury salvage, the potential utility of these miRs may still be questionable, as quick test results are required to proceed with the treatment.

### 3.2. MiRs Worth of Examination as They Might Be Prognostic for Outcomes

#### 3.2.1. miRs Predictive of Plaque Instability and Myocardial Infarction in Patients with Stable CAD or after Index ACS

While some miRs are released as a result of ischemic injury, local and systemic inflammation, others provide information on adverse cardiovascular events, risk of ACS, myocardial I/R injury, or LVR (Table 3). Data regarding the clinical meaning of miRs as prognostic biomarkers in patients with stable CAD, or following ACS are sparse, often inconsistent, and/or not systematized. They address the need for further research. Therefore, we present some findings that may stimulate future research studies with systematized methodology using optimal multicenter study designs.

Wang et al., in a cohort study of 2812 subjects of the general population, found that out of five promising serum miRs (miR-10a-5p, miR-126-3p, miR-210-3p, miR-423-3p, and miR-92a-3p), miR-423-3p was able to precisely predict cardiac events such as ACS and subsequent ACS during a median follow-up of 6 years [125]. Importantly, adding miR-423-3p to the model of traditional risk factors improved the predictive power for ACS (AUCs: 0.806 vs. 0.782) [125]. Overexpression of miR-423 has been shown to have a role in hypoxia/reoxygenation following ACS, accompanying I/R injury, and promote cardiomyocyte apoptosis [126].

Amongst platelet-derived miRs, that are responsible for platelet activation and are influenced by antiplatelet therapy, miR-223-3p is one the most investigated in the context of cardiovascular outcomes. Adding miR-223-3p level into the model for calculating ischemic risk following ACS significantly increased the predictive accuracy for CVD, as well as the combined ischemic endpoint (CVD/re-MI/IS) within 30 days and one year (Table 3) [127]. Similarly, the miR-126-3p:miR-223-3p ratio resulted in the increased predictive power for CVD and combined end-point (Table 3) [127]. In a recently published research paper, Scărlătescu et al. found that in a group of 50 young patients with recent STEMI, that echocardiographic myocardial work indices along with miR-223-3p, miR-146a-5p, and miR-142-3p were clinically utile predictors of adverse cardiovascular outcomes [128]. In this study, 18% of the STEMI patients experienced adverse events including CVD, re-admission for heart failure, or required another cardiovascular intervention at 1-year follow-up, [128]. In a study by Schulte et al. enrolling 340 ACS patients and 533 patients with stable CAD, miR-223 was observed to predict CVD in the ACS group and, after age-adjustment, also in patients with stable CAD at 4-years follow-up [129]. In ACS patients, the prognostic power of miR-223 and miR-197 was even higher [129].

In another study including 1112 secondary care patients, six miRs were able to predict the risk of CVD during a median follow-up of 4 years [130]. Ziaee et al. suggested that inflammation-related miRs, such as: miR-146a, miR-342, and miR-145 may be useful biomarkers in predictive and preventive cardiology [131]. In line with this, STEMI patients with high levels of miR-146a had a higher risk of major adverse cardiovascular events (MACE) compared to those with low miR-146a levels (log-rank *p* = 0.034). The authors suggested that miR-146a could serve as a biomarker for the adverse prognosis of STEMI and MACE at 3 years following primary ACS [132].

Whereas Bukauskas et al. found that the expression levels of miR-23a-3p were correlated with in-hospital mortality risk scores, suggesting that a down-regulation of circulating miR-23a-3p levels may be associated with an increased severity of STEMI and a higher risk of CVD [115]. It is probable that the expression levels of miR-208 can also be taken into consideration as predictors of reduced 6-month survival following ACS [133]. Finally, as one ischemic event may increase the risk for secondary ischemic events, in a study by Badacz et al., expression levels of miR-1-3p, miR-16-5p and miR-122-5p during incident ischemia were identified as risk factors of secondary cardiovascular events [134]. In addition, miR-134 was observed to be an important prognostic factor of secondary adverse cardiovascular events in patients presenting with vulnerable plaques and diabetes [135].

**Table 3:** microRNAs that may be prognostic biomarkers in stable coronary artery disease and acute coronary syndrome for cardiovascular outcomes.

Study Groups	microRNA	Down vs. Up-Regulated	Prognostic/Therapeutic	Statistical Analysis AUC, or HR/OR (95% CI), *p*-Value	Reference
Cardiovascular events					
2812 general population subjects	miR-423-3p	Up	*p*, for ACS during a median follow-up of 6 y.	Better model including miR-423 (AUC: 0.806) vs. traditional risk factors (AUC: 0.782)	Wang X., 2020 [125]
62 STEMI patients 26 healthy controls	miR-23a-3p	Down	*p*, correlates with GRACE and APACHE scores of in-hospital mortality, and 1-month survival	AUC 0.806, *p* < 0.05 *p* = 0.045 (log-rank tests)	Bukauskas T., 2019 [115]
598 ACS patients randomized to ticagrelor vs. prasugrel treatment	miR-223-3p	Up	*p*, CVD/re-MI/IS at 30d. CVD/re-MI/IS at 1 y.	15.74 (2.07–119.9), *p* = 0.008 3.18 (1.40–7.19), *p* = 0.006	Hromadka M., 2021 [127]
598 ACS patients randomized to ticagrelor vs. prasugrel treatment	miR-126 to miR-223 ratio	Low	*p*, CVD/re-MI/IS at 30d. CVD/re-MI/IS at 1 y.	0.14 (0.03–0.61), *p* = 0.009 0.37 (0.17–0.82), *p* = 0.014	Hromadka M., 2021 [127]
50 STEMI patients 10 healthy controls	miR-223-3p miR-142-3p miR-146a-5p	Up Up Up	*p*, for CVD/readmission for HF/new cardiovascular intervention	AUC 0.832, *p* =0.002 AUC 0.732, *p* = 0.031 AUC 0.848, *p* = 0.001	Scărlătescu A.I., 2022 [128]
340 ACS patients 533 patients with stable CAD	miR-197 miR-223 miR-126 miR-197 miR-223	Up Up Up Up Up	*p*, for CVD after MI at 4 y. *p*, for CVD after adjustment to age at 4 y.	2.24 (1.25; 4.01), *p* = 0.006 4.94 (1.42; 17.2), *p* = 0.012 3.47 (1.39; 8.66), *p* = 0.008 3.37 (1.35; 8.39), *p* = 0.009 3.54 (1.41; 8.92), *p* = 0.007	Schulte C., 2015 [129]
430 ACS patients 682 patients with stable CAD	miR-19b miR-132 miR-140-3p miR-150 miR-186 miR-210	Up Up Up Up Up Up	*p*, for CVD at 4 years	3.59 (1.27–10.15), 0.025 2.85 (1.33–6.08), 0.022 2.88 (1.36–6.09), 0.022 2.14 (1.21–3.79), 0.022 2.08 (1.18–3.66), 0.022 3.10 (1.12–8.55), 0.039	Karakas M., 2017 [130]
7 STEMI patients 7 healthy controls	miR-146a	Up	*p*, MACE at 3 years	1.329 (1.06–1.664), *p* = 0.01	Xiao S., 2021 [132]
21 ACS patients 8 healthy controls	miR-208b	Up	*p*, elevated miR-208b expression was associated with reduced 6-month survival	5.08 (1.13–22.82), *p* = 0.03	Alavi-Moghaddam M., 2018 [133]
142 patients with ACS or ischemic carotid event	miR-1-3p	Up	*p*, expression levels during incident ischemia are risk factors of CVD at 6 y.	2.73 (1.22–6.12), *p* = 0.014	Badacz R., 2021 [134]
Left ventricular remodeling					
80 patients with STEMI	miR-1	Up	*p*, for LVEDV increase >10% at 6 months, better value in combination with CK-MB, Nt-pro-BNP and CMR	miR-1: AUC: 0.680 miR-1 + CMR + NT-pro-BNP+ CK-MB: AUC: 0.890	Ma Q., 2020 [136]
44 ACS patients	miR-1 miR-21 miR-29a	Up Up Up	*p*, for absolute change for LVEDV at 6 months	miR-1 and miR-29b correlated with lower infarct zone, miR-29b correlated with absolute change in LVEDV	Grabmaier, U., 2017 [137]
14 patients with STEMI	miR-30a-5p	Up	*p*, for LVEF < 50%, and NT-proBNP > 150pg/mL at 6 months	AUC: 0.750 (0.58–0.92)	Maciejak A., 2018 [138]
198 ACS patients	miR-21 miR-146a	Up Up	miR-21 and miR-146a are early biomarkers of LVR *p*, for LVEDV increase >20% at 1 year	miR-146a: AUC: 0.818 miR-21: AUC: 0.719 in combination they have a higher predictive power	Liu, X., 2015 [139]
359 patients with MI	miR-34a miR-208a	Up Up	*p*, for CVD and LVEDV increase >10% at 6 months	miR-34a: OR 17.91 (2.07–98.81), *p* = 0.003 miR-208b: OR 4.18 (1.36–12.83), *p* = 0.012 combination of the two miRs: OR 18.73 (1.96–101.23), *p* < 0.001)	Lv P., 2014 [140]
113 MI patients 59 healthy controls	miR-150	Down	*p*, for HF and LVEF at 1-year, better value in combination with BNP	HR: 1.233 (1.125–1.352) miR-150 alone: AUC: 0.764 miR-150 plus BNP: AUC: 0.807	Lin X., 2019 [141]
12 ACS patients 12 healthy controls	miR-29a	Up	The greater increase in miR-29a, the greater increase in LVEDV at 90 days post MI	early miR-29a increase correlates with a negative outcome of post-MI LVR	Zile M.R., 2011 [142]
Ischemia/reperfusion injury					
44 patients deceased (19 for ACS, 25 as SCD) 18 trauma victims	miR-1 miR-499 miR-208	Up Up Up	*p*, miR-1 and miR-499 are sensitive markers to diagnose SCD compared ACS, and miR-208 for ACS vs. controls	SCD vs. ACS (AUC: 0.917) SCD vs. ACS (AUC: 0.898) ACS vs. controls (AUC: 0.855)	Pinchi E., 2019 [143]
24 patients deceased for ACS 8 patients deceased in accidents	miR-1	Up	*p*, might play role in cardiac remodeling	3.8-fold increase in miR-1 in remote myocardium	Boštjancic E., 2010 [144]
47 patients deceased for ACS, including 23 from VF 8 trauma victims	miR-133a/b	Down	*p*, may contribute to VF	For VF, 2.9-fold decrease in miR-133a/b level in remote myocardium	Boštjancic E., 2018 [145]

ACS: acute coronary syndrome; AUC: area under the curve; BNP: brain natriuretic peptide; CMR: cardiac magnetic resonance; CVD: cardiovascular death; HF: heart failure; HR: hazard ratio; IS: ischemic stroke; LVAD: left ventricular assist device; LVEDV: left ventricular end-diastolic volume; LVR: left ventricular remodeling; MACE: major adverse cardiovascular event; MI: myocardial infarction; OR: odds ratio; *p*: prognostic; SCD: sudden cardiac death; VF: ventricular fibrillation.

#### 3.2.2. miRs Associated with Myocardial I/R Injury and LVR

The most effective strategy for treating ACS is early and rapid myocardial reperfusion via percutaneous coronary intervention (PCI) [1]. However, restoring blood flow to the ischemic myocardium can induce further damage, known as I/R injury [146]. I/R contributes to increased mortality and morbidity [147]. In addition, many STEMI patients present on admission with a high-thrombus burden, which is known to carry an increased risk of distal embolization by fragmented thrombus debris [148]. This results in an increase in infarct size, as thrombotic debris constitutes a biologically active material that may exacerbate local injury via endothelial cell inflammation, higher vascular permeability, vasodilating/vasoconstricting factor imbalance, and complement and coagulation system activation [149,150].

In addition, heart failure following ischemia-induced LVR is a frequent complication of ACS. it prevalance is estimated at 30%, and is associated with a worsening of cardiovascular outcomes [151]. It accounts for 40–60% of CVD at 5 years. Besides many traditional prognostic risk factors, miRs have emerged as important biomarkers of LVR and SCD. In patients who have suffered from ACS, LVR has been associated with miR-1, miR-21, miR-29a, miR-30a, miR-34a, miR-146a, miR-150, and miR-208a expression (Table 3) [136,137,138,139,140,141,142]. Lin et al. found that out of the most common LVR-related miRs (miR-29a, -133a, -150, -192, -194, -34a, -208b, and -499), expression levels of miR-150 were observed as a good predictor for LVR and heart failure [141].

Although many cardioprotective strategies against I/R injury have been proposed, none have shown a clinically significant improvement in STEMI patients [148]. Some success has been reported with intracoronary oxygen therapy [147], or modulation of miR expression, such as miR-1, miR-21, miR-34a, and miR-146 [146,152,153,154,155].

There are only a few human studies on miR expression and the risk of I/R injury (Table 3) [143,144,145]. They include research on cardiomyocyte-derived miRs from deceased patients, such as miR-1, miR-208, miR-499 and miR-133, that are associated with extensive apoptosis, increased infarct size, and proneness to ventricular arrhythmia after ACS [143,144,145].

Unfortunately, the studied miRs differ between human and culture/animal studies. In a porcine model, the levels of miRs from the miR-15 family were increased in I/R injury, which directly promoted cardiac ischemia, while miR-15 inhibition protected against the injury [156]. In rats, up-regulated miR-21 protected against atherosclerotic growth, and protected cardiomyocytes against ACS and reactive oxygen species (ROS)-induced injury by targeting the programmed cell death 4 (PDCD4) gene [146]. Consistently, exogenous miR-21 was cardioprotective and decreased infarct size [11]. Regretfully, the clinical effect of miR-21 in individual patients with recent ACS regarding I/R injury and LVR is under discussion, as miR-21 obtains its anti-apoptotic effect through many diverse routes [157,158]. In the acute phase of ACS, overexpression of miR-21 protects against I/R injury, as decreases cardiomyocyte apoptosis [157]. However, later on, miR-21 protects fibroblasts from apoptosis, which is inconvenient as this exaggerates scarring and increases LVR [157]. Zhang et al. revealed that higher levels of miR-21 during hospitalization for ACS were associated with an increased risk of hospitalization for heart failure (Table 3) [116].

Recently, new miRs have been proposed as potential therapeutic targets after ACS [159,160,161]. In animal models, miR-19a/19b, miR-93, and miR-144 protected cardiomyocytes against I/R injury and LVR following ACS [159,160,161]. Their delivery stimulated cardiac regeneration, reduced border zone fibrosis, inflammation and apoptosis. In contrast, as miR-1 and miR-34a increase apoptosis and inflammatory response, their inhibition would be beneficial after ACS [162,163,164].

In summary, the mechanism of I/R injury and LVR are complex and multifactorial, thus the potential therapeutic approach should be a panel of microRNA-mimics and antagomirs [162,163,164,165,166,167].

## 4. Conclusions

The knowledge on miRs is constantly growing, and although not all molecular mechanisms have been recognized, miRs have entered the era of clinical application and therapy. Some miRs can be used as ‘quick’ diagnostic tests for CAD presence and severity. miR-1, miR-133, and miR-499 have the greatest potential for diagnostic biomarker of ACS, as they can be detected in blood samples before troponins. Many miRs can be used as markers of prognosis, including the widely studied miR-21 for I/R injury and LVR risk.

Much more limitations concern therapeutic approach, although miR-mimics and antagomirs are already on the board (animal studies). At different stages of atherosclerosis, different miRs can be considered for therapy, as a variety of miRs regulate particular stages of atherosclerotic development. At the beginning of atherosclerosis, a promising target is miR-142-3p that may have a role in the prevention and treatment of atherosclerosis. In addition, miR-92a seems an interesting target as it is associated with plaque progression and instability. Caution must be paid to individual miRs which role varies depending on their source or stage of atherosclerosis, e.g., miR-21, miR-155, or miR-33a/b that regulate cholesterol homeostasis and oncogene expression. Of note, single miRs can inhibit target mRNAs irreversibly, as many are very stable in circulation. Thus, some miRs could be given locally to avoid their systemic action.

In conclusion, miRs repeatedly gather support as being valuable in clinical practice as diagnostic biomarkers for CAD and ACS, prognostic biomarkers for cardiovascular outcomes, and potential therapeutic agents. Some have already been used in phase II and III clinical studies on cancer. We may expect that in a near future they will also enter into clinical practice in cardiology. The research is still ongoing.
